# The CD34-Related Molecule Podocalyxin Is a Potent Inducer of Microvillus Formation

**DOI:** 10.1371/journal.pone.0000237

**Published:** 2007-02-21

**Authors:** Julie S. Nielsen, Marcia L. Graves, Shierley Chelliah, A. Wayne Vogl, Calvin D. Roskelley, Kelly M. McNagny

**Affiliations:** 1 The Biomedical Research Centre, University of British Columbia, Vancouver, British Columbia, Canada; 2 Department of Cellular and Physiological Sciences, University of British Columbia, Vancouver, British Columbia, Canada; McMaster University, Canada

## Abstract

**Background:**

Podocalyxin is a CD34-related transmembrane protein involved in hematopoietic cell homing, kidney morphogenesis, breast cancer progression, and epithelial cell polarization. Although this sialomucin has been shown to block cell adhesion, the mechanisms involved remain enigmatic. It has, however, been postulated that the adaptor proteins NHERF-1 and 2 could regulate apical targeting of Podocalyxin by linking it to the actin cytoskeleton.

**Principal Findings:**

Here, in contrast, we find that full-length Podocalyxin acts to recruit NHERF-1 to the apical domain. Moreover, we show that ectopic expression of Podocalyxin in epithelial cells leads to microvillus formation along an expanded apical domain that extends laterally to the junctional complexes. Removal of the C-terminal PDZ-binding domain of Podocalyxin abolishes NHERF-1 recruitment but, surprisingly, has no effect on the formation of microvilli. Instead, we find that the extracellular domain and transmembrane region of Podocalyxin are sufficient to direct recruitment of filamentous actin and ezrin to the plasma membrane and induce microvillus formation.

**Conclusions/Significance:**

Our data suggest that this single molecule can modulate NHERF localization and, independently, act as a key orchestrator of apical cell morphology, thereby lending mechanistic insights into its multiple roles as a polarity regulator, tumor progression marker, and anti-adhesin.

## Introduction

The apical surface of adherent cells is a highly specialized domain that, in differentiated epithelia, is characterized by microvilli. These structures act as high surface area transport sites, and their formation coincides with the polymerization of f-actin at the core of the microvilli and recruitment of the ezrin/radixin/moesin (ERM) family of proteins to the apical domain, presumably to act as linkers between the cytoskeleton and transmembrane proteins (reviewed in [1]). Despite their clear biological importance and the striking membrane remodeling that coincides with their formation, the mechanism of microvillus assembly during epithelial morphogenesis remains poorly understood.

Podocalyxin/PCLP-1/MEP21/gp135 is a cell surface sialomucin closely related to CD34 and Endoglycan [2], [3], [4]. These three proteins each have a conserved cytoplasmic tail with a C-terminal PDZ recognition site, a transmembrane region, and an extracellular domain with extensive glycosylation, providing a bulky, negatively-charged structure [2]. Podocalyxin is a ∼140 kDa protein expressed on the surface of vascular endothelia, mesothelial cells, hematopoietic progenitors, megakaryocytes, kidney podocytes, luminal breast epithelial cells, and a subset of neurons [5], [6], [7], [8], [9], [10]. It was first identified as the major sialylated glycoprotein of renal glomerular epithelial cells (podocytes), and we have since shown that it is essential for their structure and function [2], [5]. Specifically, Podocalyxin knockout mice generate normal numbers of podocyte precursors, but they fail to generate the extensive, highly-interdigitated foot processes typical of differentiated podocytes and instead retain cell junctions between immature podocytes [2]. Thus, loss-of-function studies implicate Podocalyxin in specialized epithelial cell morphogenesis.

It has been hypothesized that the normal function of Podocalyxin is to act as an “apicalizing” factor and an anti-adhesin that can disrupt cell-cell contacts between epithelial cells, and that overexpression can lead to altered morphologies associated with cancer progression [8], [11], [12]. How these processes are regulated at the ultrastructural level, however, has not been elucidated. To define the cytosolic components that link Podocalyxin to the cytoskeleton and regulate its activity as a blocker of adhesion, a number of groups have screened for intracellular Podocalyxin-binding proteins [13], [14], [15]. This led to the identification of the extremely versatile NHERF (Na+/H+ exchanger regulatory factor) family of adaptor proteins (reviewed in [16], [17]) as Podocalyxin binding partners. NHERF-1/EBP-50 and NHERF-2/E3KARP/TKA-1 are closely related adapter proteins bearing two N-terminal PDZ domains and a C-terminal ERM domain. These proteins bind the C-terminus of a variety of membrane receptors via their PDZ domains and link them to cytoskeletal components through their conserved ERM domain. They have been implicated in regulating a wide variety of biological processes including ion transport, signal transduction, and growth control; known ligands for NHERF PDZ domains include G protein coupled receptors, receptor tyrosine kinases, and transcription factors (reviewed in [18]).

The discovery that NHERF-1 and 2 bind the C-terminal DTHL motif of Podocalyxin's cytosolic tail has led to speculation that formation of NHERF/Podocalyxin complexes may be the key mechanistic step in 1) formation of apical domains in epithelial cells, 2) dissolution of cell-cell junctions between normal epithelial cells or during tumor progression, 3) generation of highly-interdigitated foot processes for normal podocyte function, and 4) blocking hematopoietic cell adhesion [2], [8], [11], [12], [13], [15], [16]. Although it has been suggested that NHERFs are responsible for the localization of Podocalyxin in each of these scenarios, this has not been demonstrated conclusively [12], [14].

To clarify the role of Podocalyxin and NHERFs in regulating cell polarity and epithelial morphogenesis, we have ectopically expressed full-length Podocalyxin and various deletion mutants in epithelial cell lines. Strikingly, we find that Podocalyxin is a robust inducer of apical microvillus formation and that these structures recruit f-actin in a manner characteristic of normal microvilli *in vivo*. Surprisingly, however, this process required only the extracellular domain and transmembrane region of Podocalyxin; the bulk of the cytoplasmic domain including all phosphorylation sites and the highly conserved NHERF binding domain were completely dispensable for this function. When present, however, Podocalyxin's cytoplasmic tail potently recruited NHERF-1 to the apical plasma membrane; in its absence NHERF-1 was dispersed throughout the cell, suggesting that Podocalyxin may modulate NHERF function by regulating its apical membrane localization. Our results suggest that Podocalyxin is a potent regulator of epithelial cell morphogenesis, which may provide mechanistic clues to its previously described role in blocking cell adhesion and as a marker of aggressive cancers when overexpressed.

## Results

### Podocalyxin is a potent inducer of microvillus formation

**Figure 1:**
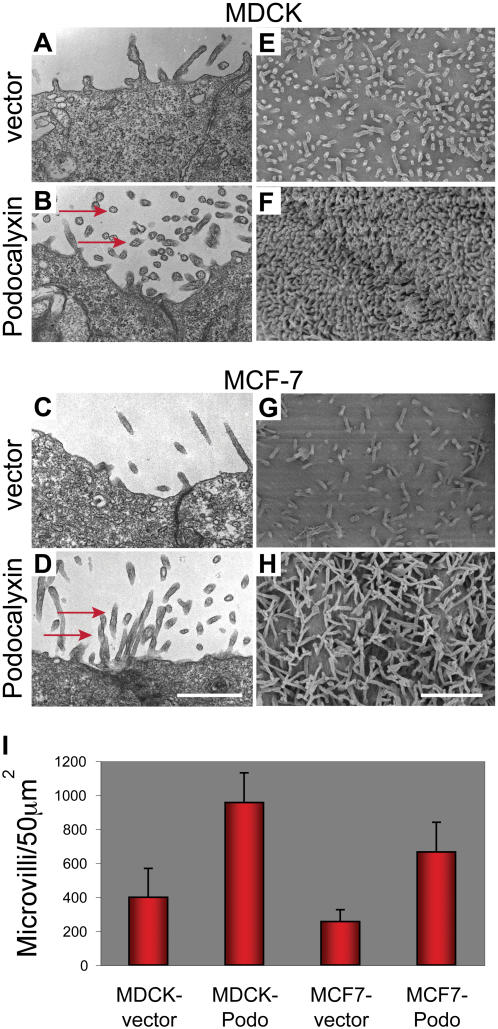
Podocalyxin induces microvillus formation. MDCK (A, B, E, F) and MCF-7 (C, D, G, H) cells transfected with murine Podocalyxin (B, D, F, H) or empty vector (A, C, E, G) and examined by TEM (A–D; scale bar: 1 µm) and SEM (E–H; scale bar: 2 µm). In TEM images, vertical slices are shown near the apical cell surface with some of the numerous additional microvilli labeled with red arrows. Many microvilli are visible as small circles as they are seen in cross-section. SEM images are of the apical cell surface with microvilli evident as thin surface projections. (I) Microvilli in six 50 µm^2^ fields were enumerated and graphed. Averages are shown; error bars represent standard deviation. T-tests were used to show statistically significant differences between vector and Podocalyxin-transfected cells, with p<0.002 in both cases. Representative of two independent experiments.

Since Podocalyxin is normally found in polarized cells with highly distinctive cell surface extensions, we investigated the possibility that this molecule may be directly involved in generation of such structures. MDCK kidney epithelial and MCF-7 breast carcinoma cell lines were stably transfected with constructs encoding full-length murine Podocalyxin and the morphologies of resulting bulk populations were examined by transmission electron microscopy (TEM). In Podocalyxin-transfected cells, but not cells transfected with empty vector, we observed a striking increase in microvillus formation (figure 1A–D). This phenomenon was even more apparent upon scanning electron microscopy (SEM) analysis of clonally isolated populations of transfected cells (figure 1E–H). Quantification revealed a 2-3-fold increase in microvillus numbers in Podocalyxin-transfected cells (figure 1I). We therefore conclude that ectopic expression of Podocalyxin is sufficient to induce the formation of microvilli.

**Figure 2:**
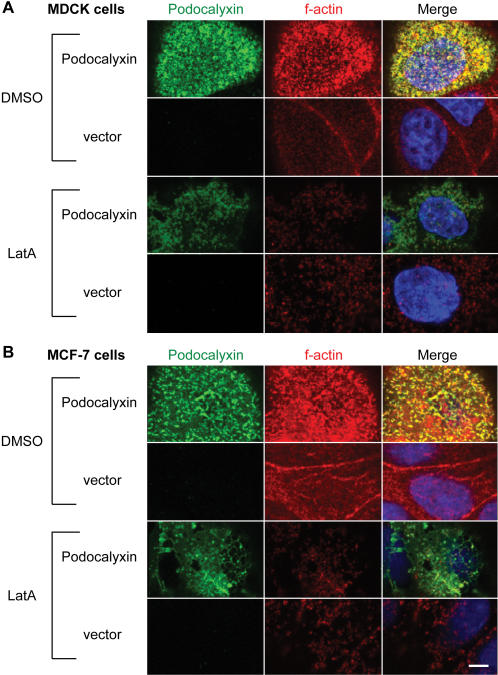
Podocalyxin-induced microvilli are structurally dependent on intact actin filaments. MDCK (A) and MCF-7 (B) cells transfected with murine Podocalyxin or empty vector were treated with the actin disrupting agent latrunculin A (LatA) or DMSO (negative control) and then fixed. Podocalyxin was labeled with an anti-Podocalyxin antibody (green); f-actin was labeled with phalloidin (red), and nuclei were labeled with DAPI (blue). Scale bar: 5 µm.

Intact actin filaments are an essential component of normal microvilli. MCF-7 and MDCK cells were therefore treated with latrunculin A, an inhibitor of actin polymerization, to determine if the Podocalyxin-induced cell surface projections were structurally dependent on f-actin. Confocal microscopic analysis revealed colocalization of f-actin and Podocalyxin in a punctate pattern at the apical cell surface where microvilli form in untreated Podocalyxin-transfected cells (figure 2). In vector-transfected cells, apical actin was more diffuse and less abundant, demonstrating that Podocalyxin is capable of apical actin recruitment, as shown previously [19]. Strikingly, latrunculin A treatment of Podocalyxin-transfected cells led to loss of actin filaments, including apically localized f-actin, and redistribution of Podocalyxin into a diffuse pattern at the cell surface. Together, these data demonstrated that Podocalyxin induces formation of typical actin-dependent microvillar structures.

**Figure 3:**
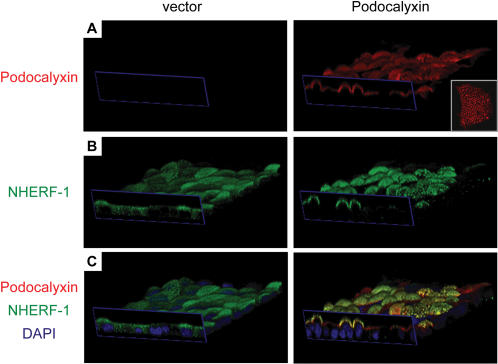
Podocalyxin induces apical expansion and recruits NHERF-1 to the apical membrane. 3D images of empty vector and Podocalyxin-transfected MCF-7 cells showing ectopic Podocalyxin (red), endogenous NHERF-1 (green), and DAPI (blue) labeling. Dimensions of blue rectangles: 118×26 µm. (A) Podocalyxin-transfected cells labeled with an anti-Podocalyxin antibody. (B) The same cells labeled with an anti-NHERF-1 antibody. (C) Merged images showing Podocalyxin, NHERF-1, and DAPI labeling. Inset in (A): projection of apical surface of a single Podocalyxin-transfected MCF-7 cell, labeled with an anti-Podocalyxin antibody.

Previously we found that Podocalyxin expression is an independent indicator of invasive breast cancer with very poor prognosis and that ectopic expression in breast carcinoma cell lines leads to subtle alterations of cell junctions and shedding of cells from confluent, high density monolayers [8]. In an effort to correlate these observations with microvillus formation, we used confocal imaging to determine the morphology and microvillus status of cells expressing Podocalyxin in monolayers. As shown in figure 3 and, more strikingly, in supplemental figure S1 [Supplementary-material pone.0000237.s002], MCF-7 cells expressing high levels of Podocalyxin exhibited a distinctive apical expansion above surrounding cells in these monolayers. This correlated closely with the appearance of a “shag-rug” morphology on the apical face of these cells as detected by Podocalyxin staining (figure 3A, inset). The same phenotype was observed in human ovarian carcinoma and normal murine mammary epithelial cell lines overexpressing Podocalyxin (data not shown). We conclude that monolayer disruption, progressive loss of cell contacts, and decreased integrity of cell junctions induced by Podocalyxin is likely linked to its ability to induce the apical domain expansion that is associated with microvillus formation.

### Podocalyxin recruits NHERF-1, f-actin, and ezrin to the apical membrane

We, and others, have shown previously that Podocalyxin can interact with NHERF-1 and NHERF-2 [12], [13], [14], [15], [19]. Since NHERF molecules act as scaffolding proteins and associate with actin through ezrin, it was assumed that NHERF proteins were responsible for apical localization of Podocalyxin. Surprisingly, here we observed the opposite. In control MCF-7 cells endogenous NHERF-1 was diffusely localized throughout the cytoplasm, whereas in Podocalyxin-transfected cells there was a dramatic increase in apically localized NHERF-1 and a concomitant decrease in cytoplasmic staining (figure 3B, C). Thus, Podocalyxin is capable of recruiting NHERF-1 to apical domains.

**Figure 4:**
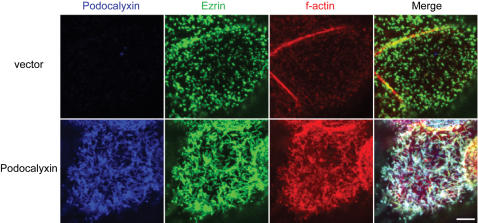
Podocalyxin induces apical recruitment of f-actin and ezrin, and all three molecules colocalize in microvilli. Projections of apical confocal images of MCF-7 cells transfected with murine Podocalyxin or empty vector showing ectopic Podocalyxin (blue), endogenous ezrin (green), and f-actin (red) labeling. White represents colocalization of all three molecules. Note the long extended microvilli in the Podocalyxin-transfected sample. Scale bars: 5 µm.

Podocalyxin and NHERF-1 both interact with the actin-binding protein ezrin [20], [21]. Since ezrin is an important component of microvilli, we sought to determine the localization of endogenous ezrin and f-actin at the apical plasma membrane in cells expressing Podocalyxin. High resolution confocal analysis revealed that in cells lacking ectopic Podocalyxin expression, ezrin localized to small discrete puncta and did not colocalize with f-actin. In contrast, in cells ectopically expressing Podocalyxin, both ezrin and f-actin were enriched at the apical membrane and dramatically colocalized with Podocalyxin in microvillar structures (figure 4). Taken together, overexpression of Podocalyxin induced the formation of apical actin-dependent microvilli, enriched with NHERF-1 and ezrin.

**Figure 5:**
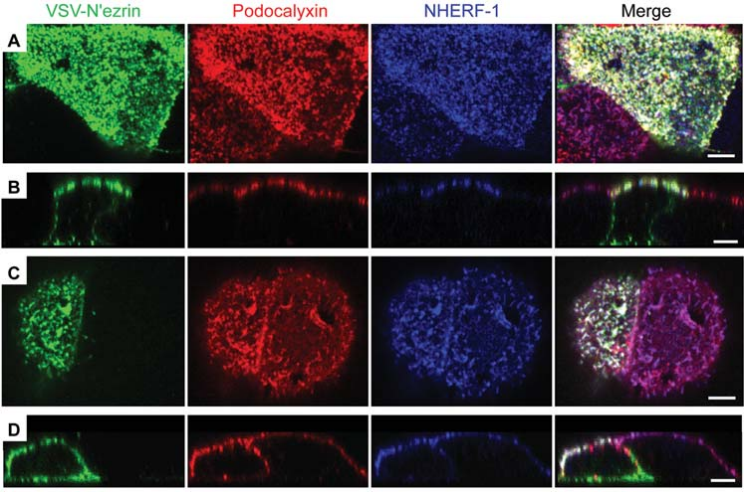
Apical localization of Podocalyxin and NHERF-1 is not dependent on ezrin. MCF-7 cells stably expressing ectopic murine Podocalyxin were either transfected with VSV-tagged dominant negative (N-terminal) ezrin after formation of monolayers and appropriate localization of Podocalyxin and NHERF-1 (A, B) or transfected with VSV-tagged N'ezrin and then replated before immunostaining (C, D). (A–D) Immunolabeling of VSV-tagged N'ezrin (green), ectopic Podocalyxin (red), and endogenous NHERF-1 (blue). Purple represents apical colocalization of Podocalyxin and NHERF-1 in cells not expressing dominant negative ezrin (internal negative control); white represents apical colocalization of all three molecules in cells expressing dominant negative ezrin. Scale bars: 5 µm. (A, C) Projections of merged confocal stacks taken near the apical cell surface showing individual colors and merged images; (B, D) vertical slices of confocal stacks.

Since ezrin is known to link transmembrane proteins to the actin cytoskeleton, we assessed the role of ezrin in the recruitment of Podocalyxin to the apical membrane by transiently expressing a dominant-negative mutant of the molecule (N'ezrin [22]) in MCF-7 cells already stably expressing Podocalyxin (figure 5). When N'ezrin was expressed in pre-formed, fully polarized monolayers, Podocalyxin and NHERF-1 both remained localized at the apical membrane (figure 5A, B). A considerable amount of the N'ezrin itself was also targeted to the apical domain in these cells (figure 5A) although, as expected [22], the mutant protein was also targeted to basal and basolateral domains (figure 5B). We also expressed N'ezrin in cells prior to monolayer formation, which allowed us to examine protein localization as polarity was being established (figure 5C, D). In this case N'ezrin was clearly present at apical, lateral, and basal surfaces (figure 5D). The same was not true of Podocalyxin and NHERF-1, which were found mainly apically and laterally as polarity was being established. Importantly, there was no difference in Podocalyxin and NHERF-1 localization in neighboring cells that did not express mutant N'ezrin. Thus, while we cannot rule out subtle differences in localization, it appears that ezrin is not likely required for Podocalyxin's apical targeting or its ability to recruit NHERF-1 to the apical domain and retain it there. This does preclude the possibility, however, that Podocalyxin may be capable of regulating the localization of endogenous, wild type ezrin (see figures 4 and 9).

**Figure 6:**
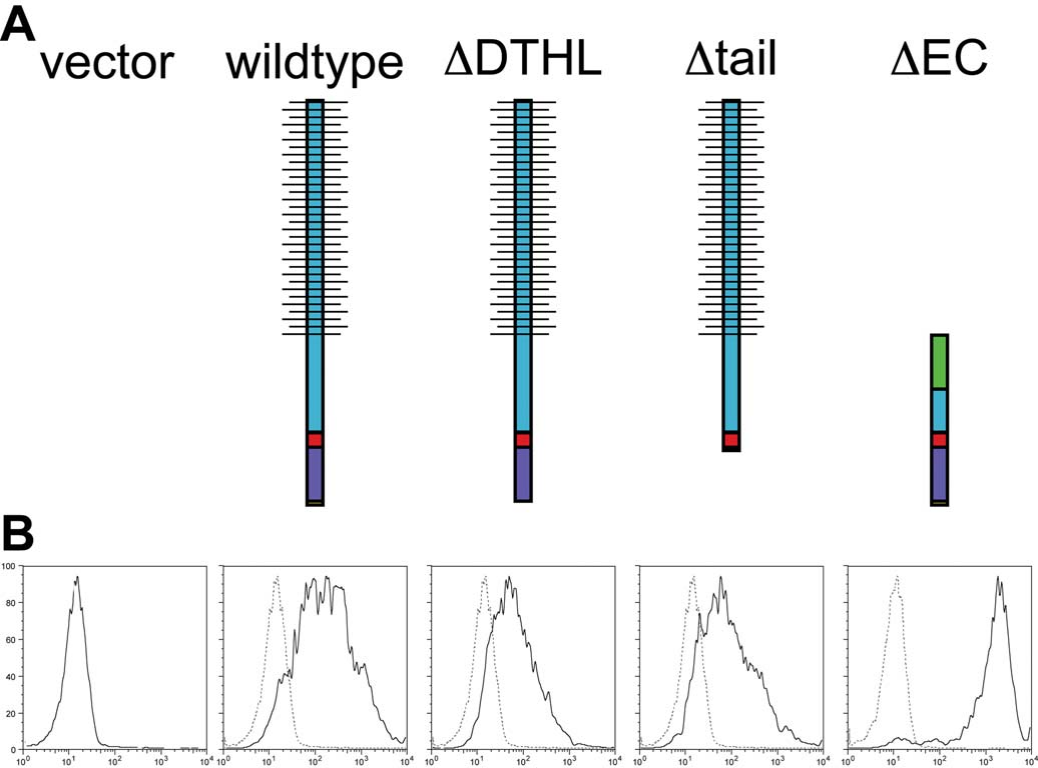
Schematic of Podocalyxin mutants and FACS expression profiles. (A) Podocalyxin mutants. Blue: extracellular domain, horizontal bars: carbohydrates/sialic acid residues, red: transmembrane region, purple: cytoplasmic tail (including C-terminal DTHL in wildtype and ΔEC), and green: flag-tag replacing most of Podocalyxin's extracellular domain. (B) FACS profiles demonstrating comparable Podocalyxin expression levels in sorted clones. Dashed lines represent negative controls (vector-transfected cells stained with the same antibodies); solid lines represent Podocalyxin expression. Note: all mutants were detected with an anti-Podocalyxin antibody with the exception of ΔEC, which was detected with an anti-flag antibody.

**Figure 7:**
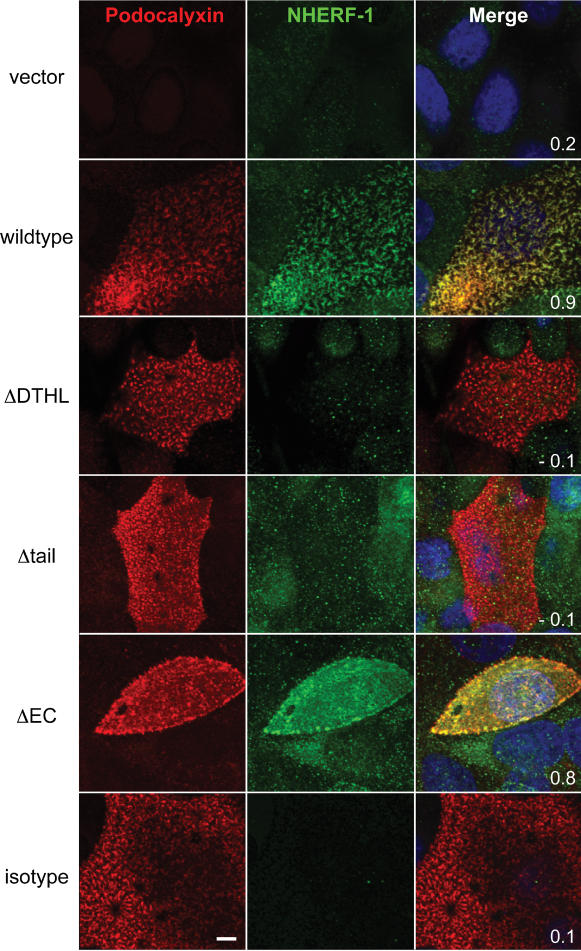
Podocalyxin's C-terminal DTHL motif is required for recruitment of NHERF-1. Projections of apical confocal images of transfected MCF-7 cells showing ectopic Podocalyxin (red), endogenous NHERF-1 (green), and DAPI (blue) labeling. Isotype control sample is Podocalyxin-transfected cells labeled with anti-Podocalyxin (red) and NHERF-1 isotype control (green) to demonstrate lack of non-specific NHERF-1 staining. White numbers represent Pearson's colocalization coefficient. Scale bar: 5 µm.

**Figure 8:**
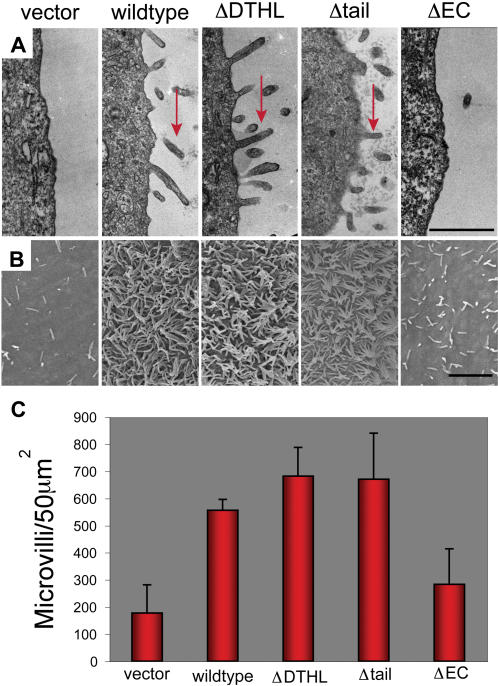
Podocalyxin's extracellular domain and the first four amino acids of its cytoplasmic tail are necessary and sufficient for microvillus formation. (A) TEM images of MCF-7 cells transfected with vector or full-length and mutant Podocalyxin. Vertical slices are shown near the apical cell surface with some of the numerous additional microvilli labeled with red arrows. Scale bar: 1 µm. (B) SEM images at the apical surface of transfected MCF-7 cells with microvilli evident as thin surface projections. Scale bar: 2 µm. (C) Microvilli in six 50 µm^2^ fields were enumerated and graphed. Averages are shown; error bars represent standard deviation. T-tests were used to show statistically significant differences between vector and Podocalyxin-transfected cells, and between wildtype Podocalyxin and ΔEC-transfected cells with p<0.003 in all cases. Representative of two independent experiments.

**Figure 9:**
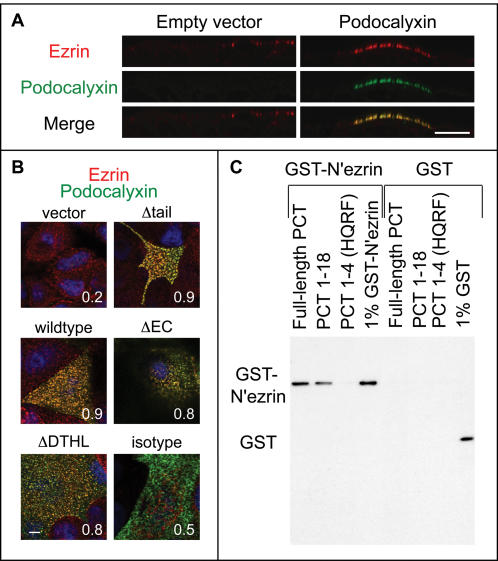
Podocalyxin colocalizes with ezrin in a manner independent of any direct interaction with NHERF-1. (A) Vertical slices of confocal stacks demonstrate increased apical recruitment of ezrin in Podocalyxin-transfected MCF-7 cells. Green: ectopic Podocalyxin, red: endogenous ezrin, blue: nuclei (DAPI). Scale bar: 5 µm. (B) Projections of merged apical confocal images of MCF-7 cells transfected with various Podocalyxin constructs or empty vector. Green: Podocalyxin, red: ezrin (or isotype control for ezrin), blue: DAPI. White numbers represent Pearson's colocalization coefficient. Scale bar: 5 µm. (C) *In vitro* pull-down assays with biotinylated Podocalyxin cytoplasmic tail (PCT) peptides and GST-N'ezrin reveal a lack of interaction between a Podocalyxin peptide representing the cytoplasmic tail of the Δtail mutant (HQRF) and ezrin. Biotinylated Podocalyxin peptides bound to streptavidin-sepharose were incubated with GST-N'ezrin or GST alone, and bound recombinant proteins were detected with an anti-GST-antibody by Western blot.

In order to gain further insights into the mechanisms involved in Podocalyxin-induced NHERF-recruitment and microvillus formation, we generated a series of MCF-7 clones expressing wild-type avian Podocalyxin or the following three deletion mutants: 1) “ΔDTHL,” lacking the C-terminal DTHL motif essential for interaction with NHERF proteins [14], [15]; 2) “Δtail,” lacking the entire cytoplasmic tail with the exception of the juxtamembrane sequence HQRF, which was retained as a membrane anchor (this mutant lacks all potential phosphorylation sites, the C-terminal NHERF-binding site, and a recently described ezrin-binding site) [3], [4], [19], [23]; and, finally, 3) “ΔEC,” lacking the majority of the extracellular domain (including the mucin domain and the cysteine-bonded globular domain) and instead encoding an extracellular “flag-tag,” Podocalyxin's transmembrane region, and its full-length cytoplasmic tail (figure 6A). Avian Podocalyxin was chosen as the basis for these experiments for three reasons: 1) it is 85% identical to mammalian Podocalyxin in its intracellular domain, 2) as with the murine protein used in the above experiments, wildtype avian Podocalyxin also induced microvillus formation in MCF-7 cells, and 3) ectopic expression could be selectively detected using a species-specific monoclonal antibody that reacts with native, fixed, and denatured forms of the molecule [3]. MCF-7 cells were transfected with these constructs, and clones were isolated based on similar levels of ectopic Podocalyxin expression as determined by FACS (figure 6B).

As shown in figure 7, full-length Podocalyxin showed strong colocalization with endogenous NHERF-1 at the apical membrane, while ΔDTHL and Δtail mutants, both of which lack the C-terminal NHERF binding motif, did not. In contrast, the ΔEC mutant lacking the extracellular domain but retaining the entire cytoplasmic tail of Podocalyxin was apically targeted and colocalized with NHERF-1. This further demonstrates that Podocalyxin recruits NHERF-1 to the apical domain through its C-terminal DTHL motif.

### Although the cytoplasmic domain is dispensable, the extracellular domain of Podocalyxin is required for microvillus formation

We then assessed how loss of the Podocalyxin/NHERF-1 interaction affected microvillus formation. MCF-7 cells transfected with the Podocalyxin mutants described above were used for analysis of microvillus formation by TEM and SEM. TEM demonstrated that, as expected, control cells had few microvilli while cells transfected with full-length Podocalyxin displayed a dramatic increase in microvillus number (figure 8A). Strikingly, cells expressing ΔDTHL or Δtail mutants generated microvilli in similar numbers to full-length Podocalyxin transfectants. Conversely, the extracellular domain of Podocalyxin was found to be essential for this phenotype since there was no increase in microvilli in cells expressing the ΔEC mutant bearing only the flag-tagged transmembrane region and the entire cytoplasmic domain of Podocalyxin, even though this mutant was able to apically recruit NHERF-1 (see figure 7, above). This result was confirmed via SEM analysis (figure 8B). In order to quantitate this effect, all microvilli observed in six random 50 µm^2^ (15 000×) SEM fields were enumerated for each mutant (figure 8C). Full-length, ΔDTHL, and Δtail transfectants all had at least twice as many microvilli as vector-control and ΔEC transfectants. We therefore conclude that the extracellular domain, transmembrane region, and four amino acids of Podocalyxin's cytoplasmic tail are sufficient to induce NHERF-1-independent formation of epithelial microvilli.

It has been shown that maintenance of podocyte foot process integrity is critically dependent on the negatively-charged glycosylations decorating Podocalyxin's extracellular domain [5], [24], [25]. It was therefore not surprising that deletion of the majority of Podocalyxin's extracellular domain also prevented microvillus formation in MCF-7 cells. In order to more precisely assess the role of sialic acid residues in microvillus formation, SEM analysis was performed on Podocalyxin-transfected MCF-7 cells after treatment with neuraminidase to remove these residues from Podocalyxin's mucin-like domain. Unexpectedly, however, treatment with neuraminidase either before or after microvillus formation did not affect the level of cell surface protrusions (data not shown).

### Microvillus formation and recruitment of ezrin and f-actin occur in the absence of NHERF-binding

It was surprising that the highly conserved, NHERF-binding, cytoplasmic tail of Podocalyxin was dispensable for microvillus formation. In broad terms, it is known that the integrity of the actin cytoskeleton is essential for maintaining cell shape, and that linkage of actin to the plasma membrane is important for generating and supporting cell surface protrusions, including microvilli [26]. Since NHERF-1 and 2 can connect Podocalyxin to the actin cytoskeleton through ezrin [13], [15], [27], we initially assumed that Podocalyxin's involvement in the formation of microvilli would require interaction with NHERFs. Moreover, NHERF-2 interactions with Podocalyxin have recently been shown to coincide closely with the formation of a “pre-apical domain” in MDCK cells, and it was postulated that this association was a prerequisite for the formation of this domain [12]. However, our data suggest that although the C-terminal tail of Podocalyxin is sufficient to actively recruit NHERF-1 to apical plasma membrane domains, the formation of microvilli and, indeed, the apical targeting of Podocalyxin to the plasma membrane, both occur in the absence of direct interaction with NHERF-1 (MCF-7 cells do not express appreciable amounts of NHERF-2, data not shown).

Interestingly, all forms of Podocalyxin, regardless of whether or not they contained a NHERF-binding sequence, displayed increased apical recruitment of endogenous ezrin and strong colocalization with this protein (figure 9A, B). Thus, recruitment of wild type ezrin to the apical membrane can also occur in the absence of any direct interaction of Podocalyxin with NHERF.

Schmieder *et al* recently demonstrated, however, that the juxtamembrane region of Podocalyxin's cytoplasmic tail can interact directly with ezrin, and that the histidine, arginine, and serine residues in the juxtamembrane **H**Q**R**I**S** sequence of rat Podocalyxin are particularly important for this interaction [19]. This sequence is similar to the ezrin-binding site in the juxtamembrane region of intercellular adhesion molecule (ICAM)-3's cytoplasmic tail [19], [28]. Although the binding of our Podocalyxin Δtail mutant (with the cytoplasmic tail HQRF) directly to ezrin remained a formal possibility, Serrador *et al* have shown that truncation of ICAM-3's cytoplasmic tail after the REHQRSGS sequence completely abolishes ezrin binding [28]. To confirm that our truncation eliminated any residual ezrin binding, we performed *in vitro* binding assays using biotinylated peptides corresponding to our Δtail mutant (HQRF), a longer peptide (HQRFSQKKSQQRLTEELQ) containing the previously described ezrin binding site, and the full-length Podocalyxin tail [19], [28]. Although we obtained reproducible binding of ezrin to the two longest peptides, we did not observe any interaction between ezrin and our truncated peptide (figure 9C) or a control peptide containing amino acids 53–73 of the Podocalyxin cytoplasmic tail (data not shown). Thus, although several important amino acids are retained in the Δtail mutant, a longer sequence is apparently required for directly interacting with ezrin. In summary, our results suggest that apical recruitment of ezrin and the formation of microvilli were not dependent on direct interaction of Podocalyxin with ezrin or on indirect interaction through NHERF proteins.

**Figure 10:**
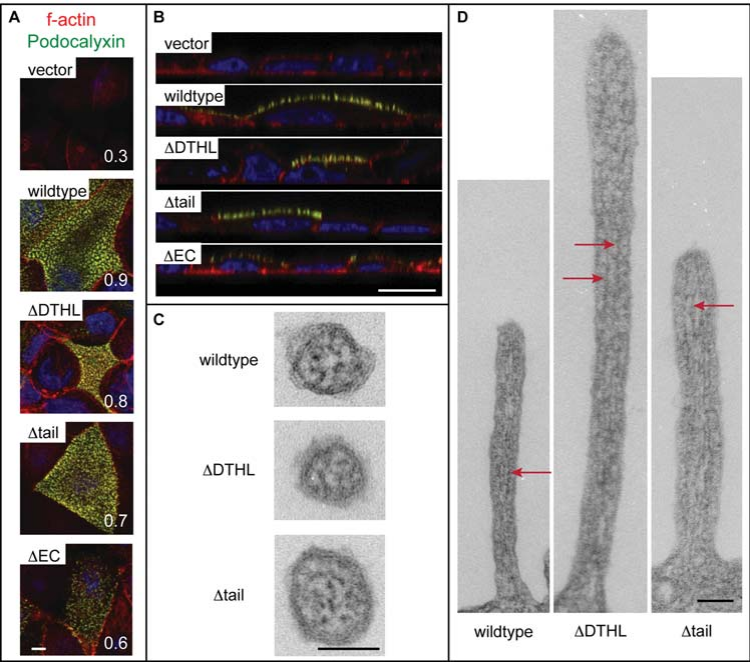
Podocalyxin colocalizes with f-actin at the apical surface of cells, even in the absence of most of Podocalyxin's cytoplasmic tail. (A, B) Podocalyxin-transfected MCF-7 cells with f-actin (red), ectopic Podocalyxin (green), and DAPI (blue) labeling. Scale bars: 5 µm. (A) Projections of merged apical confocal images; white numbers represent Pearson's colocalization coefficient. (B) Vertical slices of confocal stacks. (C, D) High magnification TEM images demonstrate the presence of actin filaments (red arrows) running the length of individual microvilli. Although microvilli of different lengths are shown in this figure, there was no consistent difference in microvilli lengths between samples. Scale bars: 0.1 µm. (C) Cross-sections; (D) longitudinal-sections.

Apical recruitment of f-actin was also independent of NHERF-binding or direct interaction of Podocalyxin with ezrin. All forms of Podocalyxin demonstrated apical colocalization with f-actin, although apical recruitment was notably less robust in the absence of the extracellular domain (figure 10A, B). Consistent with the SEM and TEM data, cells expressing extracellular-domain-containing Podocalyxin mutants exhibited a clear punctate staining pattern on the apical surface of cells in protruding structures indicative of microvilli (figure 10A, B). To confirm that these structures bear all the structural hallmarks typical of microvilli, they were examined at high magnification by TEM. As with full-length Podocalyxin, ΔDTHL and Δtail mutants each clearly demonstrated the presence of actin filaments in the microvillar core (figure 10C, D). In summary, Podocalyxin is able to recruit actin to microvilli in the absence of the bulk of its cytoplasmic domain, but the extracellular domain is essential for this process.

## Discussion

### Podocalyxin's role in determining cellular morphology

Experiments from the 1970's suggested that highly-glycosylated and sialylated glycoproteins play a key role in maintaining the integrity of the foot processes of kidney podocytes [24], [25], [29]. With the subsequent identification of Podocalyxin as the major component of the podocyte glycocalyx and the tight correlation between its expression and podocyte morphogenesis *in vivo*, this molecule became the prime candidate as a regulator of foot process formation [5], [30], [31], [32]. Our gene targeting studies confirmed this: although Podocalyxin deficient animals generate podocyte precursors, these cells fail to undergo morphogenesis [2]. Moreover, this defect is rescued by kidney-specific ectopic expression of Podocalyxin (Nielsen *et al*, in preparation). In the current experiments, we extend these observations and find that rather than being a podocyte-specific phenomenon, expression of Podocalyxin is sufficient for induction of morphogenesis and microvillus formation in epithelial cell lines. Importantly, endogenous Podocalyxin is normally expressed and apically targeted in mammary epithelium [8] and the ovarian surface epithelium (J. Cipollone and C. Roskelley, unpublished observations) *in vivo* suggesting that this represents a normal biological process.

Podocalyxin's activity as a cell morphogen is dependent on its extracellular domain since mutants lacking this domain, though apically targeted, are unable to form microvilli. Several previous studies *in vivo*, though indirect, support this notion. For example, treatment of kidney podocytes to neutralize the negatively charged sialic acid residues on the podocyte surface leads to a dramatic loss of podocyte interdigitating foot processes [24], [25], [29], [33]. It is therefore not surprising that deletion of the majority of Podocalyxin's extracellular domain also prevents microvillus formation. A caveat with this model, however, is our inability to detect a loss of microvilli in neuraminidase-treated cells suggesting that, at least for microvillus formation, the terminal sialic acid residues are dispensable. This discrepancy could reflect differences in 1) the cellular context of Podocalyxin expression (podocytes versus other epithelia), 2) the size of the structures formed (foot processes versus microvilli), or 3) the microenvironment of the treated cells (*in vivo* kidney podocytes versus cultured breast epithelial cells). Supporting these observations, it has been noted that neuraminidase treatment of kidney podocytes *in vivo* leads to a disruption of the interaction between Podocalyxin and ezrin while treatment of Podocalyxin expressing MDCK cells does not [15], [21]. Importantly, it has recently been demonstrated that mice bearing a glycosyltransferase mutation that prevents terminal glycosylation of Podocalyxin have a much milder phenotype than the complete Podocalyxin knockout that we previously generated [2], [34]. Thus, further work will be required to determine the degree to which specific glycosylations regulate Podocalyxin's ability to orchestrate microvillus assembly.

**Figure 11:**
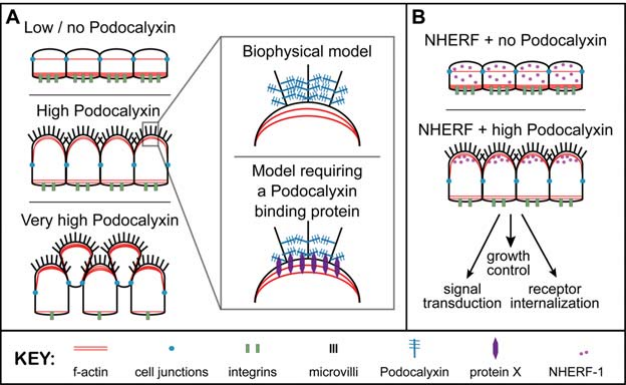
Model of Podocalyxin's functions. (A) Cells expressing low or no Podocalyxin have well-defined cell-cell junctions and adhere well to the substratum. Overexpression of Podocalyxin leads to recruitment of f-actin to the apical membrane for formation of microvilli along with apical domain expansion and weakening of cell-cell junctions. Higher expression induces further recruitment of f-actin to the apical surface, which may lead to a decrease in basolateral actin and disruption of integrin-mediated cell-substratum adhesion. Two models are proposed to explain Podocalyxin's mechanism of action. (B) In the absence of Podocalyxin expression NHERF-1 is localized throughout the cytoplasm, whereas in cells expressing high levels of Podocalyxin NHERF-1 is recruited to the apical membrane. Thus, Podocalyxin may regulate NHERF's role in many biological processes.

Although the precise mechanism by which Podocalyxin induces microvillus formation is unclear, it may be largely biophysical; the formation of microvilli may simply serve as a mechanism to distribute Podocalyxin's bulky extracellular domains more evenly across the cell surface. In this model, an increasing amount of cell surface Podocalyxin would lead to an increasing expansion of the apical domain of these cells (figure 11A). Continued expression would lead to marginalization of cell-cell junctions and, eventually, a decrease in cell-cell contact and adhesion. This model is consistent with the previous reports of a dose-dependent weakening of cell junctions induced by ectopic expression of Podocalyxin and the tight correlation between Podocalyxin overexpression and breast cancer metastatic index [8], [11]. It is also consistent with recent reports of Podocalyxin's role as a “preapical” domain-forming protein [12].

While the overexpression of certain actin-associated intracellular proteins, such as espin and villin, have long been known to stimulate microvillus formation [35], [36], it has also been shown recently that some transmembrane proteins can induce similar phenotypes. In ovarian follicle cells, overexpression of Cad99C (the Drosophila orthologue of the human Usher cadherin PCDH15) increases microvillus length, and Cad99C-null flies have shortened microvilli [37]. Similarly, overexpression of syndecan-3 or the mucin, podoplanin, leads to filopodia formation [38], [39], [40]. Thus, Podocalyxin is not the only transmembrane protein that can induce formation of cell surface extensions. It is also intriguing that, like Podocalyxin, podoplanin has been proposed to play a functional role in metastatic carcinoma progression by altering polarization rather than by initiating a classical epithelial to mesenchymal transformation [8], [39].

A more unique aspect of the present studies is the apparent dispensability of Podocalyxin's cytoplasmic domain, since microvillus formation is known to be associated with apical recruitment of f-actin and ERM proteins. Deletion of the cytoplasmic domain of syndecan-3, for example, dramatically reduces the number of filopodia on transfected cells [40]. Similarly, although much of the cytoplasmic portion of Cad99C can be deleted without affecting microvillus length, the residual 31 intracellular amino acids may contain recognition sites for cytoplasmic adapter molecules or cytoskeletal components [37]. Thus, we provide the first example demonstrating the absolute necessity of an extracellular domain, in the absence of a cytoplasmic domain, in formation of microvilli. In addition, our results demonstrate that Podocalyxin-dependent formation of microvilli does not require direct interaction with NHERF proteins (detailed further below) [12], [14], [15], [16], [19].

### How does Podocalyxin induce membrane projections?

Although we have shown that the extracellular and transmembrane domains are sufficient for generation of microvilli, there are several possible mechanisms that could lead to this effect (figure 11A). As stated above, Podocalyxin could have a passive or biophysical role involving apical membrane expansion. In this scenario epithelial cells would cope with this Podocalyxin-dependent apical domain expansion (possibly as a consequence of a unique property of its extensive glycosylation) by passively creating membrane projections that are subsequently locked into place by the linkage of other apically targeted proteins to the actin cytoskeleton. Alternatively, we favor a model in which Podocalyxin could play a more active role in microvillus production. Podocalyxin's extracellular domain could directly bind another interacting transmembrane molecule that is able to recruit ezrin and f-actin and induce direct production of microvilli rather than passive apical domain expansion. This interaction may therefore transduce the signal to initiate microvillus formation. Regardless of the model, the retention of the microvillus formation phenotype in the presence of neuraminidase suggests that this specific glycosylation is not a prerequisite for this phenotype. Instead Podocalyxin's stalk or globular domain, or perhaps the remainder of its bulky mucin-like domain, may be particularly important in this interaction (under investigation).

### What is the significance of NHERF recruitment to apical domains by Podocalyxin?

Several groups have suggested that NHERF proteins are responsible for strict apical localization and membrane retention of Podocalyxin [12], [14], [19]. In these studies, deletion of Podocalyxin's NHERF docking site led to a modest reduction in apical Podocalyxin. Although we agree that loss of the NHERF binding site may lead to a subtle relocalization of a small portion of Podocalyxin, our studies demonstrating complete relocalization of NHERF-1 from the cytoplasm to the apical membrane when full length Podocalyxin is ectopically expressed argue that it is primarily Podocalyxin that regulates the distribution of NHERF-1 (and presumably other NHERF-bound signaling molecules) rather than NHERF-1 regulating the distribution of Podocalyxin. Although our results preclude a direct functional role for NHERF/Podocalyxin interactions in formation of microvilli and apical cell domains, apical recruitment of NHERF-1 by Podocalyxin is likely to be very important for other aspects of NHERF function, which include ion transport, signal transduction, growth control, receptor signaling, and receptor internalization. Regarding the latter, NHERF can bind a wide variety of signaling ligands (ie. EGFR, PDGFR, β-catenin, PTEN, and adrenergic and purinergic receptors, to name but a few) with functional consequences [18], [41]. The fact that Podocalyxin is a potent inducer of NHERF recruitment to microvilli may suggest a new link between the formation of these specialized structures and these diverse biological processes. Regardless, the dramatic Podocalyxin-dependent recruitment of NHERF-1 to the apical plasma membrane likely affects NHERF-1's activity as a signaling scaffold (figure 11B).

### Podocalyxin and microvilli in cell adhesion and cancer

In most situations Podocalyxin appears to act as an anti-adhesion molecule [2], [11]. For example, we have shown that Podocalyxin expression is upregulated in metastatic breast cancer cells and that it induces delamination of these cells from monolayers [8]. Given our present results, it is not unreasonable to suggest that this function may be closely linked to Podocalyxin's ability to generate microvilli. Specifically, Podocalyxin-coated, microvilli-rich apical domains on free surfaces may protect cells from non-specific adhesion to extracellular matrices, regardless of the polarization state of the cells. Thus, Podocalyxin overexpression in breast carcinomas may promote tumor cell dissemination by initiating a general disruption of cell adhesion, particularly under conditions where apical membrane domains are expanded due to breakdown in polarity. Such a novel mechanism of invasive tumor cell dissemination, which has also been proposed for podoplanin [39], may be clinically important in the most prevalent ductal breast carcinoma subtype as these tumors rarely undergo dissemination by a recognizable epithelial to mesenchymal transformation [42], [43].

It is also interesting to speculate that the recruitment of f-actin to the apical membrane for microvillus formation might deplete actin from the basal surface of these cells [19] and thereby prevent stable interactions between integrins and the extracellular matrix. In ovarian carcinoma cells this may be the case as Podocalyxin overexpression decreases integrin recruitment to the cell-extracellular matrix interface (J. Cipollone and C. Roskelley, unpublished observations). This hypothesis is further supported by the observation that cells transfected with full-length Podocalyxin or mutants lacking its cytoplasmic tail generate abundant microvilli and exhibit decreased cell-substratum adhesion, while cells transfected with Podocalyxin lacking the extracellular domain do not generate microvilli and remain adherent ([8], figure 11A, and unpublished observations). Future studies delineating the mechanisms by which apical Podocalyxin and basolateral integrins compete for actin will clarify this process.

## Materials and Methods

### Antibodies

Primary antibodies were mouse monoclonals against ezrin (biotinylated 3C12: NeoMarkers, Fremont CA and purified 3C12: Abcam, Cambridge UK), chicken Podocalyxin (Mep21) [44], the VSV epitope for detecting VSV-tagged N'ezrin (P5D4: Sigma-Aldrich, Oakville ON), and the flag epitope for detecting the flag-tagged ΔEC mutant (biotinylated M2: Sigma-Aldrich), a rat monoclonal against mouse Podocalyxin (MBL, Woburn MA), and polyclonal rabbit antibodies against NHERF-1 (EBP50: Abcam) and GST (Santa Cruz Biotechnology, Santa Cruz, CA). Secondary antibodies were Alexa Fluor 488 conjugated goat anti-mouse or rabbit IgG, Alexa Fluor 568 conjugated goat anti-mouse IgG_1_, rat IgG, or streptavidin, Alexa Fluor 647 conjugated goat anti-rat or rabbit IgG (Invitrogen, Burlington ON), biotinylated goat anti-rat IgG (Southern Biotech, Birmingham AL), APC conjugated goat anti-mouse Ig or streptavidin (BD Biosciences, Mississauga ON), and horseradish peroxidase conjugated goat anti-rabbit Ig (DAKO, Mississauga, ON). Rabbit IgG (Jackson ImmunoResearch Laboratories, Westgrove PA) and biotin-conjugated mIgG_1_ (R&D Systems, Minneapolis MN) were used as isotype controls. F-actin was detected with Alexa Fluor 568 or rhodamine conjugated phalloidin (Invitrogen); DAPI was used for nuclear staining (Sigma).

### DNA Constructs and Podocalyxin mutants

Murine Podocalyxin cDNA was a generous gift from Dr. David Kershaw; chicken Podocalyxin cDNA was cloned from HD100 hematopoietic progenitor cells [3]. Podocalyxin mutants were generated by PCR from cDNA using primers designed to insert early stop codons after the juxtamembrane HQRF sequence (Δtail) or before the C-terminal DTHL (ΔDTHL). The ΔEC mutant was generated by replacing Podocalyxin's mucin and globular extracellular sequences with CD43's signal peptide and a flag-tag motif, generously provided by Wooseok Seo and Dr. Hermann Ziltener. PCR products were cloned into TOPO (Invitrogen), then pIRES2-EGFP (BD Biosciences), and sequenced in the final constructs. VSV-tagged dominant negative (N-terminal) ezrin was a generous gift from Dr. Monique Arpin.

### Cell culture, transfection, and cell sorting

MCF-7 human breast carcinoma and MDCK canine kidney epithelial cells were routinely maintained in Advanced D-MEM/F12 medium (Invitrogen #12634-010) supplemented with 5% fetal bovine serum, penicillin, streptomycin, and glutamine. Some experiments were performed in both high and low serum to assess whether these conditions altered protein localization or microvillus formation, but we concluded that there was no difference. Cells were transfected with 30 µg pIRES2-EGFP (BD Biosciences), or the same vector containing Podocalyxin cDNA, using the DMRIE-C transfection reagent (Invitrogen). Stable transfectants were selected by culturing in 400 µg/ml G418 and sorted for GFP or Podocalyxin-positive bulk populations or clones using a BD FACS Vantage. For actin disruption experiments, transfected cells were treated with 10 µm latrunculin A (Sigma-Aldrich) in media for 10 minutes before fixation. For dominant negative ezrin experiments, cells stably expressing ectopic murine Podocalyxin were transiently transfected with VSV-tagged N'ezrin, replated 12 hours post-transfection or not replated, and fixed 36 hours post-transfection.

### Electron microscopy

Cells were grown on filters (TEM) or glass coverslips (SEM), fixed, and processed for electron microscopy. Images were collected using a Philips 300 electron microscope (TEM) or a Hitachi S4700 FESEM. Figures were arranged using Adobe Photoshop and Adobe Illustrator software. Microvilli were enumerated in six random 15 000×(50 µm^2^) fields for each sample. Statistical significance was demonstrated using T-tests to compare vector versus Podocalyxin-transfected cells (or wildtype Podocalyxin versus ΔEC-transfected cells in the case of the ΔEC mutant).

### Immunofluorescence microscopy

Cells were grown on glass coverslips, fixed with 4% paraformaldehyde, permeabilized with 0.1% Triton X-100, labeled with primary and secondary antibodies (described above), and examined using an Olympus Fluoview FV1000 confocal microscope. Triple-labeled images were collected sequentially. Micrographs were generated with several merged confocal planes or vertical sections of confocal stacks using Olympus Fluoview FV1000 software (version1.3b). Pearson's coefficient was used to quantitate colocalization on individual slices using ImagePro 3DS 6.0 software. Photos were arranged with Adobe Photoshop and Adobe Illustrator software.

### *In vitro* binding assay using purified recombinant proteins

Biotinylated Podocalyxin tail peptides were produced with a spacer and the following sequences: HQRF (the cytoplasmic tail of the Δtail mutant), HQRFSQKKSQQRLTEELQ (partial tail of Podocalyxin including the ezrin binding site), and the full-length Podocalyxin cytoplasmic tail. A pGEX construct encoding the N-terminal domain of ezrin fused with GST was a generous gift from Dr. Monique Arpin. GST and GST-N'ezrin were produced according to manufacturer's instructions (Amersham Biosciences, Piscataway, NJ). Biotinylated Podocalyxin peptides (4.3 nmol) were bound to streptavidin-sepharose (Amersham Biosciences), and then incubated for 2 hours at 4°C with 20 µg GST or an equimolar amount of GST-N'ezrin in 150 mM NaCl, 20 mM Tris (pH7), and 0.2% Triton X-100 containing 1 mM PMSF and a protease inhibitor cocktail (Sigma-Aldrich). Bound proteins were detected by immmunoblotting using an anti-GST antibody.

## Supporting Information

Video S1A Podocalyxin induces apical domain expansion and NHERF-1 recruitment. 3D rotated reconstructions of MCF-7 cells transfected with (A) empty vector or (B) murine Podocalyxin showing Podocalyxin (red), NHERF-1 (green), and DAPI (blue) labeling. The 3D images were generated from confocal stacks and re-sliced down the Z axis every 5 μM along the Y axis using Olympus FluoView1000 imaging software. The resulting movies were modified using ImagePro Plus Discovery 3D (Media Cybernetics).
(26.65 MB AVI)

Video S1B Podocalyxin induces apical domain expansion and NHERF-1 recruitment. 3D rotated reconstructions of MCF-7 cells transfected with (A) empty vector or (B) murine Podocalyxin showing Podocalyxin (red), NHERF-1 (green), and DAPI (blue) labeling. The 3D images were generated from confocal stacks and re-sliced down the Z axis every 5 μM along the Y axis using Olympus FluoView1000 imaging software. The resulting movies were modified using ImagePro Plus Discovery 3D (Media Cybernetics).
(25.73 MB AVI)
